# Recognition of multimolecular G-quadruplex regulates phase separation of cockayne syndrome B

**DOI:** 10.1093/nar/gkag708

**Published:** 2026-07-15

**Authors:** Naura Fakhira Antariksa, Michele Stasi, Marco Di Antonio

**Affiliations:** Department of Chemistry, Molecular Sciences Research Hub, Imperial College London, 82 Wood Lane, London, W12 0BZ, United Kingdom; Institute of Chemical Biology, Molecular Sciences Research Hub, Imperial College London, 82 Wood Lane, London, W12 0BZ, United Kingdom; Department of Chemistry, Molecular Sciences Research Hub, Imperial College London, 82 Wood Lane, London, W12 0BZ, United Kingdom; Institute of Chemical Biology, Molecular Sciences Research Hub, Imperial College London, 82 Wood Lane, London, W12 0BZ, United Kingdom; Department of Chemistry, Molecular Sciences Research Hub, Imperial College London, 82 Wood Lane, London, W12 0BZ, United Kingdom; Institute of Chemical Biology, Molecular Sciences Research Hub, Imperial College London, 82 Wood Lane, London, W12 0BZ, United Kingdom; The Francis Crick Institute, 1 Midland Road, London, NW1 1AT, United Kingdom

## Abstract

Cockayne syndrome B (CSB) is a multifaceted protein with known functions in DNA repair and transcription elongation. We recently demonstrated that CSB can recognize DNA secondary structures known as multimolecular G4s (mG4s) with high selectivity, but the potential biological significance of this interaction remains to be elucidated. In this study, we report that CSB can undergo liquid–liquid phase separation (LLPS), a process heavily linked with transcriptional regulation. Importantly, we found that the binding of CSB to mG4s promotes LLPS, leading to the physical segregation of DNA sequences containing mG4s within CSB–mG4 droplets from those lacking this structural motif. Furthermore, we revealed that mG4-binding alters the physicochemical properties of the phase-separated CSB, including increased salt resistance and decreased in-droplet mobility. Given the growing evidence supporting an active role for G4s in stimulating transcription, we anticipate that the selective LLPS displayed by CSB upon mG4 binding may be relevant in the context of transcriptional regulation.

## Graphical Abstract

**Graphical Abstract:**
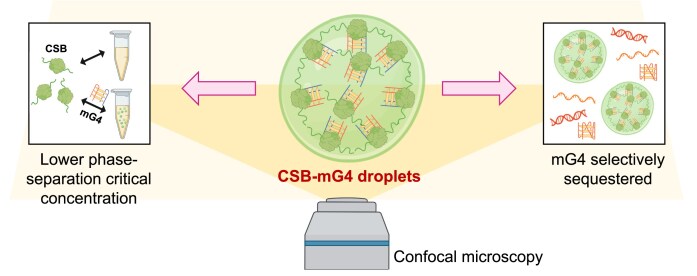

## Introduction

Cockayne syndrome B (CSB) protein is a key regulator of transcription-coupled nucleotide excision repair and transcription elongation [1, 2]. This protein acts as an ATP-dependent translocase; it facilitates the forward movement of stalled RNA polymerase II [3–6], particularly due to transcriptional blockages and helix-distorting DNA damages. Indeed, mutations in this protein lead to Cockayne syndrome disease, an incurable disease with an extremely poor prognosis [2, 7].

**Figure 1.**
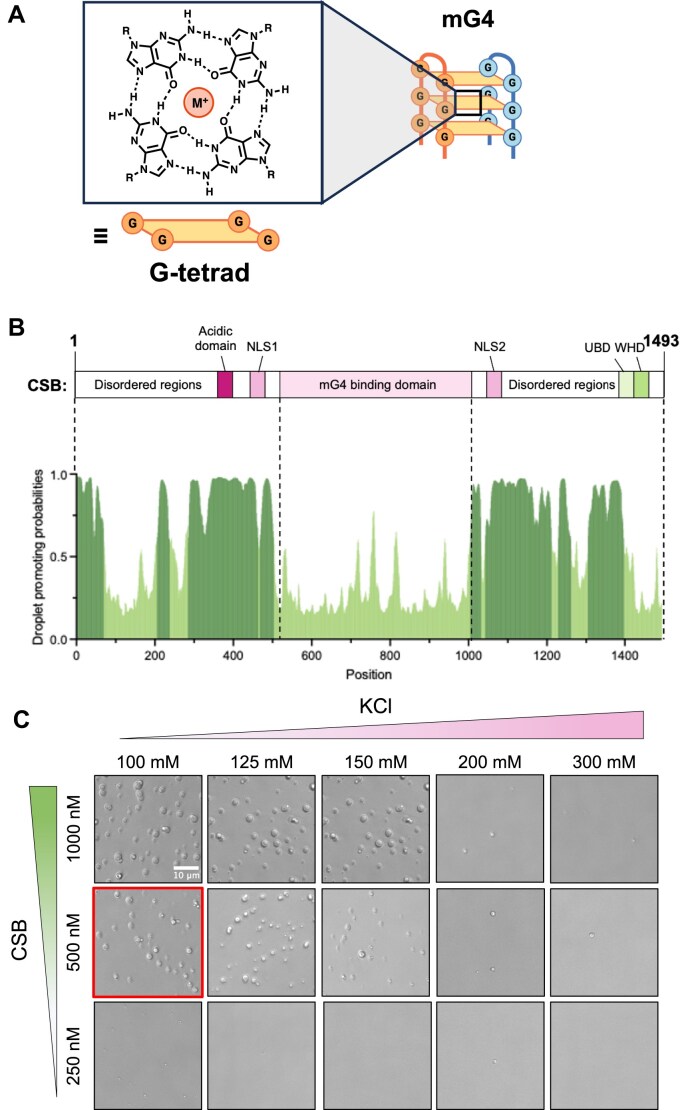
Liquid–liquid phase separation (LLPS) of CSB was observed *in vitro* at physiological conditions. (**A**) A schematic illustration of a dimeric mG4s; Image Created in Biorender, Robinson, J. (2026) https://BioRender.com/5zd51ti. (**B**) FuzDrop prediction depicting potential phase-separating regions in the CSB sequence. (**C**) Brightfield confocal micrographs demonstrating phase separation of CSB under different conditions [(CSB) = 250–1000 nM; (KCl) = 100–300 mM]; scale bar = 10 μm. Droplet formation is increased at higher protein concentration and lower ionic strength (KCl).

While the role of CSB in facilitating transcription and DNA repair is well characterized, our recent work has revealed that CSB can also interact with DNA secondary structures known as G-quadruplexes (G4s) (Fig. 1A) [8, 9]. G4s are formed through Hoogsteen G-G base pairings to generate a planar tetramer (G-tetrad), which is further stabilized by coordination of a monovalent cation (in order of stability K^+^ > Na^+^ > Li^+^). These structures have been implicated in various biological processes, including telomere maintenance [10], DNA replication [11], and transcription [12–14]. G4s can form within a single-strand DNA sequence (unimolecular) or across multiple distinct DNA sequences (multimolecular). However, multimolecular G4s (mG4s) have not been considered biologically relevant, as the formation of multivalent distal interactions across distinct DNA sequences has been deemed unlikely to occur in cells. Nevertheless, our recent finding revealed that CSB exhibits exceptional binding affinity and selectivity for mG4s compared to the more-studied unimolecular G4s (uG4s) [8]. This observation raises the intriguing possibility that mG4 recognition plays a relevant role in the transcriptional functions elicited by CSB, especially considering the documented enrichment of G4s in the regulatory regions of actively transcribed genes [12, 13, 15–19].

CSB canonically resides within transcription factories [20, 21], which have previously been described as biomolecular condensates potentially formed through LLPS [22–24]. Biomolecular condensates sequester macromolecules within membrane-free compartments, increasing local protein concentration and promoting transcription [25]. Previous studies have already linked G4s to LLPS [26–31], suggesting an active role of these structures in the formation and regulation of biomolecular condensates. In this work, we set out to elucidate whether CSB’s selective recognition of mG4s is associated with the formation of biomolecular condensates, which would reconcile the known role of CSB in transcription with its affinity for mG4s.

## Materials and methods

### Materials

Unless stated otherwise, chemicals were purchased from Sigma–Aldrich, VWR, and ThermoFisher Scientific and used without further purification. All reagents were of molecular biology grade. Oligonucleotides were purchased from IDT; non-labeled oligonucleotides were purified via standard desalting, whereas fluorophore-labeled nucleotides were purified via HPLC.

### Generation of recombinant high-titer virus for CSB purification

pFASTBac plasmid encoding for the HA-CSB-His_6_ construct was donated by the Scheibye-Knudsen group. The plasmid was transformed into DH10Bac cells for blue/white screening. White colonies were selected, and the bacmid DNA was purified from 2 ml of *Spodoptera frugiperda* Sf9 cells at 1 × 10^6^ cells/ml, which were seeded in six-well plates and transfected with 500 ng of the bacmid. Supernatant P1 was harvested after 5 days, and 400 μl was used to infect 25 ml of Sf21 cells at 1 × 10^6^ cells/ml. After 5 days, when the cell viability had dropped below 80%, the supernatant P2 was harvested. Four microliters of P2 was used to infect 25 ml of Sf21 cells at 1 × 10^6^ cells/ml. After 5 days, when cell viability had dropped below 80%, the supernatant P3 was harvested and used for large-scale protein purification.

### Expression and purification of CSB

12 × 550 ml of Sf21 suspension culture at 1.5 × 10^6^ cells/ml was prepared in roller bottles. Two milliliters of P3 virus was added to infect the cells, followed by incubation at 27°C, shaking at 150 rpm for 72 h. The cells were then harvested by centrifugation at 2000 *g* for 30 min.

Pellet resulting from 3-l culture (~30 ml) was resuspended in 70 ml ice-cold H_2_O, followed by the addition of 100 ml 2× lysis buffer (100 mM Tris, pH 7.5, 1600 mM NaCl, 20 mM imidazole, pH 8, 20% glycerol, 20 mM MgCl_2_, 2 mM TCEP) supplemented by 4 protease-inhibitor tablets (cOmplete™, EDTA-free Protease Inhibitor Cocktail, Roche) and 20 μl Universal nuclease (Thermo Scientific). The cells were disrupted with one pass through the Emulsiflex cell homogenizer. Lysate was clarified by centrifugation at 25 000 RPM for 60 min in a Beckman-Coulter Ti 50.2 fixed-angle rotor. The soluble fraction was loaded onto 5 ml TALON Crude column (Cytiva) using Buffer A (50 mM Tris, pH 7.5, 800 mM NaCl, 10 mM imidazole, pH 8, 10% glycerol, 1 mM TCEP) as wash buffer and washed further with Buffer B (50 mM Tris, pH 7.5, 200 mM NaCl, 20mM imidazole, pH 8, 10% glycerol, 1 mM TCEP). Bound proteins were eluted in Buffer B supplemented with 400 mM imidazole (pH 8).

Fractions containing CSB were pooled and diluted with H_2_O (~15 ml protein sample + 5 ml H_2_O) and loaded onto a 5 ml HiTrap Heparin HP column (Cytiva) equilibrated in Buffer C (50 mM Tris, pH 7.5, 150 mM NaCl, 2 mM MgCl_2_, 1 mM TCEP). CSB was eluted using a linear NaCl gradient from 0 to 1000 mM. The protein was eluted at a conductivity of around 50 mS/cm (beginning of the peak) to 70 mS/cm (end of the peak).

Fractions containing CSB were pooled and concentrated to 2 ml in a Vivaspin PES 30 kDa MWCO (Sartorius) and applied to a HiLoad^®^ 16/600 Superdex^®^ 200 pg (Cytiva) size-exclusion chromatography column at a flow-rate of 1 ml/min in 50 mM Tris, pH 7.5, 200 mM NaCl, 1 mM TCEP, 2 mM MgCl_2_.

### Fluorescent labeling of CSB protein

Twenty microliters of CSB protein (1 mg/ml) was thawed and supplemented with 1 mM TCEP (pH 7.5). The protein was incubated at room temperature for 20 min to allow the reduction of cysteine residues by TCEP. Meanwhile, Cy3-maleimide (Lumiprobe, 11080) stock solution was prepared in dimethyl sulfoxide by resuspending the dye to a concentration of 1 mg/ml (1.5 mM). A five-fold molar excess of Cy3-maleimide was added to the CSB protein, followed by incubation in the dark at room temperature for 120 min. Excess fluorescent dye was removed using a Zeba™ Spin Desalting Column (MWCO 7 kDa; Thermo Scientific) per the manufacturer’s instructions. The protein was stored in 50 mM Tris (pH 7.5), 200 mM NaCl, 2 mM MgCl_2_, 1 mM TCEP, 10% glycerol buffer, flash-frozen in liquid nitrogen, and stored at −80°C until use.

### General oligonucleotide preparation

Oligonucleotides were prepared at a 5:95 labeled-to-unlabeled ratio for all microscopy experiments, except partitioning, where a 1:1 ratio was utilized. Labeled Cy5-rDNA oligonucleotide was used as is for gel analysis and electrophoretic mobility shift assays (EMSA). The oligonucleotides were prepared to a final concentration of 2 μM in a buffer containing 25 mM HEPES (pH 8.0), 100 mM KCl. The oligonucleotides were then annealed by heating at 95°C for 3 min, followed by slow cooling to room temperature at a rate of −1°C/min.

### Confocal fluorescence microscopy

All microscopy experiments were performed on µ-Plate 384 Well Glass Bottom (Ibidi, 88407). A Leica SP8 confocal microscope was used to image droplets on the plate. 40× oil immersion objectives were used, and 1 Airy unit was set for the pinhole. Droplets containing oligonucleotides labeled with the Cy5 dye were excited at 633 nm (HeNe laser) and signal acquired from 655 to 685 nm. Those containing oligonucleotides labeled with the FAM dye were excited at 488 nm and detected at 500–530 nm. The protein, which was stained with SYPRO^TM^ Orange (Thermo Fisher) or covalently labeled with Cy3, and oligonucleotides labeled with Cy3 were imaged with a 514 nm excitation laser and detected at 550–585 nm. The PMT detector was utilized for all experiments.

Images were recorded at 1024 × 1024 pixels, 2× zoom, and a scan speed of 400 Hz. Z-stacks were recorded at 512 × 512 pixels (for Z-stack), 2× zoom, and 600 Hz scan speed. Measurements were performed at room temperature, and the resulting images were analyzed using ImageJ software.

### Sample preparation for microscopy experiments

All reactions were prepared in 10 μl reaction mixtures, combining 2.5 μl of 4× reaction buffer (80 mM HEPES, pH 8.0, 160 ng/μl bovine serum albumin (BSA), 4 mM dithiothreitol (DTT), 4 mM MgCl_2_, *varying concentration of KCl*) at various concentrations of protein, salt, and oligonucleotides:

- For salt and protein titration experiments, KCl concentrations were varied between 100 and 300 mM (final concentration), whereas CSB protein concentrations were varied between 250 and 1000 nM. The oligonucleotide concentration was kept at 500 nM.
- In oligonucleotide titration experiments, the final KCl concentration was maintained at 100 mM, and the protein concentration at 100 nM. Oligonucleotides were added at varying concentrations from 0 to 1000 nM.
- Unless stated otherwise, samples for fluorescence recovery after photobleaching (FRAP) and partitioning experiments were prepared in a reaction buffer with 100 mM KCl, and a 500 nM equimolar amount of oligonucleotide and CSB.
- In experiments to displace the CSB–mG4 interaction with CX-5461 or AppNHp (Jena Bioscience), the final KCl concentration was maintained at 100 mM, whereas the protein and oligonucleotides were added at an equimolar concentration of 500 nM. CX-5461 or AppNHP was added to a final concentration of 1 mM.

After mixing all reagents in PCR tubes, the reactions were incubated at 30°C for 30 min, after which the tubes were placed on ice for 10 min. SYPRO™ Orange (Thermo Fisher) was added at a final concentration of 0.8× to facilitate protein staining when CSB–Cy3 was not utilized.

### Fluorescence recovery after photobleaching

A Leica SP8 confocal microscope was used to perform all FRAP experiments on a 384-well plate (Ibidi, 88407). Cy5 was bleached using a 633 nm laser and imaged at 655–685 nm with a PMT detector. The pinhole was set to 1 Airy unit, and images were recorded at 512 × 64 pixels, 2.5× zoom, and a scan speed of 700 Hz. Recovery data were corrected for photobleaching and background as follows:


\begin{eqnarray*}
{{I}_{\mathrm{corrected}}}\left( t \right) = \frac{{{{I}_{\rm raw}}\left( t \right) - {{I}_{\rm BG}}\left( t \right)}}{{{{I}_{\mathrm{fading}}}\left( t \right) - \ {{I}_{\rm BG}}\left( t \right)}},
\end{eqnarray*}

where ${{I}_{\mathrm{corrected}}}( t )$ is corrected fluorescence intensity at time point *t*, ${{I}_{\rm raw}}( t )$ is raw fluorescence intensity at time point *t*, ${{I}_{\rm BG}}( t )$ is fluorescence intensity of background at time *t*, and ${{I}_{\mathrm{fading}}}( t )$ is fluorescence intensity of non-bleached droplet in the same field of view at time point *t*.

The corrected intensities were normalized by the prebleach intensity as follows:


\begin{eqnarray*}
{{I}_{\mathrm{normalized}}}\left( t \right) = \frac{{{{I}_{\mathrm{corrected}}}\left( t \right)}}{{{{I}_{\mathrm{prebleach}\_AVG}}\left( t \right)}},
\end{eqnarray*}

where ${{I}_{\mathrm{normalized}}}( t )$ is normalized fluorescence intensity, and ${{I}_{\mathrm{prebleach}\_AVG}}$ is average fluorescence intensity before photobleaching.

The normalized intensity was then further normalized to the lowest value as follows:


\begin{eqnarray*}
{{I}_{\rm DN}}\left( t \right) = \frac{{{{I}_{\mathrm{normalized}}}\left( t \right) - \rm MIN}}{{\rm MAX - \rm MIN}},
\end{eqnarray*}

where ${{I}_{\rm DN}}( t )$ is doubly normalized fluorescence intensity, $\rm MIN$ is the minimum intensity value on the trace, and $\rm MAX$ is the maximum intensity value on the trace.

The normalized FRAP traces were fitted to the one-phase association binding model ($Y = {{Y}_0} + ( {\mathrm{Plateau} - {{Y}_0}} )( {1 - {{e}^{ - Kx}}} )$ using GraphPad Prism 10.3.0, from which the recovery half-time was extracted.

The mobile fraction was calculated using the following formula:


\begin{eqnarray*}
{\rm MF} = \frac{{{{I}_{{\mathrm{final}}}} - {{I}_0}}}{{{{I}_{{\mathrm{prebleach}}}}\left( t \right) - {{I}_0}}},
\end{eqnarray*}

where mobile fraction, ${{I}_{\mathrm{final}}}$ is final recovered intensity and ${{I}_0}$ is intensity after photobleaching.

### Oligonucleotide gel analysis

Unlabeled oligonucleotides were prepared to varying final concentrations (1–10 μM) in a buffer containing 25 mM HEPES (pH 8.0), 100 mM KCl. The oligonucleotides were then annealed by heating at 95°C for 3 min, followed by slow cooling to room temperature at a rate of −1°C/min. The annealed oligonucleotides were diluted to 500 nM in a 10 μl solution to mirror the experimental conditions for microscopy. The samples were resolved on a 10% native polyacrylamide gel (with a 4% stacking gel) at 60 V for 10 min, followed by 80 V for 90 min in 1× Tris-borate–EDTA (TBE; Invitrogen) at 4°C. The gel was then stained with NMM (10 μg/ml in 1× TBE supplemented with 100 mM KCl) for 10 min and imaged using LI-COR Odyssey^**®**^ M imaging system, set on the EtBr channel. The gel was then destained in 1× TBE supplemented with 100 mM KCl for 1 h, upon which the gel was stained with SYBRGold (Invitrogen) for 30 min. The gel was visualized using the LI-COR Odyssey^**®**^ M imaging system, set to detect SYBRGold.

### CSB electrophoretic mobility shift assay

Ten microliters of EMSA reaction mixtures were prepared by combining 2.5 μl of 4× reaction buffer (80 mM HEPES, pH 8.0, 160 ng/μl BSA, 4 mM DTT, 4 mM MgCl_2_, 400 mM KCl), annealed Cy5-labeled rDNA-mG4 to a final concentration of 25 nM, and CSB protein to a final concentration of 50 nM. AppNHP (Jena Bioscience) was added at an increasing concentration from 0 to 10 mM. These mixtures were incubated at 30°C for 30 min, after which the reaction was quenched by adding 2 μl of no-SDS purple gel-loading dye (New England Biolabs). The samples were resolved on an 8% native PAGE gel (with a 4% stacking gel) at 60 V for 10 min, followed by 80 V for 90 min in 1× TBE (Invitrogen) at 4°C. The gels were visualized using the LI-COR Odyssey^**®**^ M imaging system, set to detect Cy5. All of the experiments were performed in biological triplicate.

### Statistical methods

**Figure 2.**
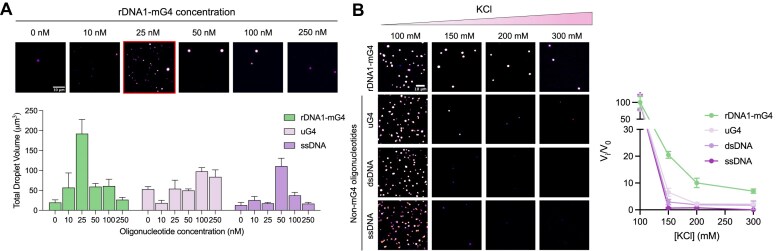
Interaction with mG4 alters the phase separation behavior of CSB. (**A**) Top: Confocal fluorescence microscopy images showing that the addition of rDNA1-mG4 to CSB (100 nM) allows phase separation to occur at a concentration below the saturation concentration for droplet formation; scale bar = 10 μm. Bottom: Comparison with the titration of other oligonucleotides. (**B**) Left: KCl dependence of CSB-oligonucleotide droplets at 500 nM equimolar concentrations of CSB and each respective oligonucleotide; scale bar = 10 μm. Right: Quantification of the differential drop in droplet volume (V_0_ = total droplet volume at 100 mM KCl, V_i_ = total droplet volume at a given KCl concentration) among CSB-oligonucleotide condensates. Error bars are shown for the standard deviation. Volume calculations for each condition were obtained from three field-of-views.

Droplet volume calculations for each condition in the phase boundary and KCl titration experiments were obtained from three to four field-of-views. For the KCl titration experiment (Fig. 2C), the total droplet volume at a given KCl concentration (V_i_) was normalized to the total droplet volume at 100 mM KCl (V_0_). For each condition, an average was determined, and error bars were shown for the standard deviation. For partitioning experiments, the partitioning constant (*K*_P_) for each condition was obtained from three to four field-of-views. Statistical significance was assessed using a paired *t*-test, with *P* < .05 considered significant. For FRAP experiments, data were obtained from 4–5 measurements per condition, with error bars indicating the standard deviation for each data point. For droplet disruption experiments, droplet diameter was measured as described above, and the average droplet diameter was calculated from three field-of-view measurements per condition. The statistical significance of the result was determined using two-way ANOVA with the Tukey correction, with *P* < .05 considered significant.

## Results

### CSB phase separates

Proteins bearing intrinsically disordered domains (IDRs) can readily undergo LLPS through their molecular flexibility and ability to form multivalent interactions [25]. Using the computational prediction software, FuzDrop [32–34], we analyzed CSB’s sequence propensity to phase separation and aggregation. This analysis showed that the N- and C-termini of CSB display high disorder, making them potential drivers for the formation of biomolecular condensates (Fig. 1B). In particular, we identified the presence of stretches of disorder-promoting residues (e.g. proline, glycine, glutamic acid) [33, 35], combined with a depletion of order-promoting residues (e.g. tryptophan, phenylalanine) in residues 1–503 and 1003–1400 of the protein. Conversely, the catalytic domain, which also serves as the mG4-binding pocket, is highly structured and unlikely to drive phase separation.

To experimentally assess the ability of the protein to undergo LLPS, we prepared 1000 nM CSB in a buffer containing a physiological salt concentration (100 mM KCl, Fig. 1C). Confocal microscopy revealed that CSB can form distinct phase-separated droplets in the absence of crowding agents at a concentration as low as 250 nM (Fig. 1C). We observed that lower protein concentrations result in smaller droplets, which is reflected by the decrease in average diameter (Supplementary Fig. S1A). This suggests that CSB’s phase separation properties are dependent on the concentration of the protein itself. To identify the supramolecular interactions that drive CSB phase separation, we exposed the droplets to two sets of physicochemical perturbations: increasing ionic strength (KCl 100–300 mM KCl) and hexanediol (0%–5% v/v) [36–38]. We observed that increasing the ionic strength of the buffer strongly affects the protein’s phase separation, leading either to a substantial reduction in droplet numbers or to their complete dissolution (Supplementary Fig. S1B). In contrast, when CSB was exposed to 1,6-Hexanediol, we detected no significant change in droplet size or number (Supplementary Fig. S1C). 1,6-Hexanediol is typically used to disrupt weak hydrophobic interactions, as droplets formed through electrostatic interactions have been shown to be insensitive to hexanediol treatment [39, 40]. This suggests that CSB phase separation is mainly driven by electrostatic interactions, likely through oppositely charged residues in the protein’s IDR, rather than hydrophobic interactions [41].

### Interaction with mG4 facilitates droplet formation at lower CSB concentrations

Given that CSB can selectively bind to mG4s, we asked whether phase-separated CSB droplets could incorporate mG4 structures through direct binding. To achieve this, we prepared fluorescently tagged CSB through labeling with Cy3-maleimide (Supplementary Fig. S2). We then used a solution of CSB (1 μM containing 5% of the tagged protein) and incubated it at 30°C for 30 min with 500 nM Cy5-labeled mG4-forming sequences derived from ribosomal DNA (rDNA1-3) (Supplementary Table S1), which has previously been characterized as a high-affinity binding substrate for CSB [8]. These sequences are the cognate substrate of CSB [9, 42] and are shown to form mG4s under the experimental conditions for microscopy (Supplementary Fig. S3A). All three sequences are polymorphic and display different distributions of mG4 species. As shown in Supplementary Fig. S3B, mG4 sequences were readily recruited into CSB condensates, suggesting that CSB droplets can actively recruit mG4s.

We then investigated whether the recruitment of mG4s within CSB droplets could affect the phase separation properties of the protein itself, as protein–nucleic acid interactions are known to promote droplet formation [43, 44]. To assess this, we prepared a CSB solution at 100 nM in physiological buffer (100 mM KCl), which is below the saturation concentration for droplet formation, ensuring that no droplets were formed. Upon addition of rDNA1-mG4 sequences, CSB droplet formation was observed in a concentration-dependent manner, reaching a maximum global droplet volume of ~200 μm^3^ at 25 nM of the oligonucleotide (Fig. 2A top). As expected, further increasing the oligonucleotide concentration led to droplet dissolution due to increased ionic strength in the solution (Fig. 2A, bottom).

Similar observations were made upon addition of rDNA2-mG4 and rDNA3-mG4 sequences, which also form stable mG4s (Supplementary Fig. S4A). Interestingly, we observed that rDNA3-mG4 exacerbates droplet formation, allowing a maximum droplet volume to be reached at 10 nM of oligonucleotide concentration, which likely reflects a better mG4-folding efficiency by rDNA3.

We then tested whether other DNA structures could also promote CSB droplet formation (Supplementary Fig. S3). When testing both double-stranded DNA (dsDNA) and *c-MYC* uG4, we observed that higher oligonucleotide concentrations (100 nM for uG4 and 50 nM for dsDNA) were required to drive droplet formation (Supplementary Fig. S4B). However, under these conditions, fewer droplets were formed (maximum droplet volume for CSB-rDNA1-mG4 ~200 μm^3^, CSB-rDNA2-mG4 ~200 μm^3^, CSB-rDNA3-mG4 ~120 μm^3^, CSB-uG4 ~100 μm^3^, CSB-dsDNA ~110 μm^3^; Fig. 2A bottom, Supplementary Fig. S4A bottom). Taken together, these findings suggest that the specific interaction between CSB and mG4s can promote CSB phase separation at lower protein concentrations. Given that mG4s are multimeric structures, we reasoned that they could increase the effective valency of CSB’s network, thereby justifying the observed phase boundary shift [45, 46]. Non-mG4 species, which are not directly bound by CSB [8], are limited to electrostatic interactions and therefore affect phase separation to a significantly lower extent.

To further investigate this, we also measured the resistance of CSB droplets formed upon binding to mG4 at increased ionic strength (Fig. 2B, left). At a 1:1 stoichiometry of CSB and rDNA1-mG4 (500 nM), droplet formation was observed under physiological conditions (100 mM KCl). Increasing the KCl concentration resulted in a progressive decrease in the number of droplets, with ~10% remaining at 300 mM KCl (Fig. 2B, right). CSB droplets formed with rDNA2-mG4 and rDNA3-mG4 exhibit comparable resistance to changes in ionic strength (Supplementary Fig. S5). In contrast, CSB droplets formed in the presence of non-mG4 oligonucleotides fully dissolved at 150 mM KCl concentration. We also utilized a scrambled rDNA1 sequence (rDNA1s) as an additional control for a non-mG4-forming sequence (Supplementary Fig. S5), which dissolves at 150 mM KCl, suggesting that the structural features rather than the oligonucleotide composition of mG4 are responsible for the observed behavior. The enhanced salt resistance further indicates increased structural organization of the CSB network upon binding to mG4s, which results in a significant improvement in droplet stability [26, 47]. Additionally, the structural order provided by the specific mG4-binding may increase charge density, thereby conferring stronger electrostatic contact with CSB and allowing for more robust condensates [26].

### mG4 Binding alters the physical properties of the droplets

**Figure 3.**
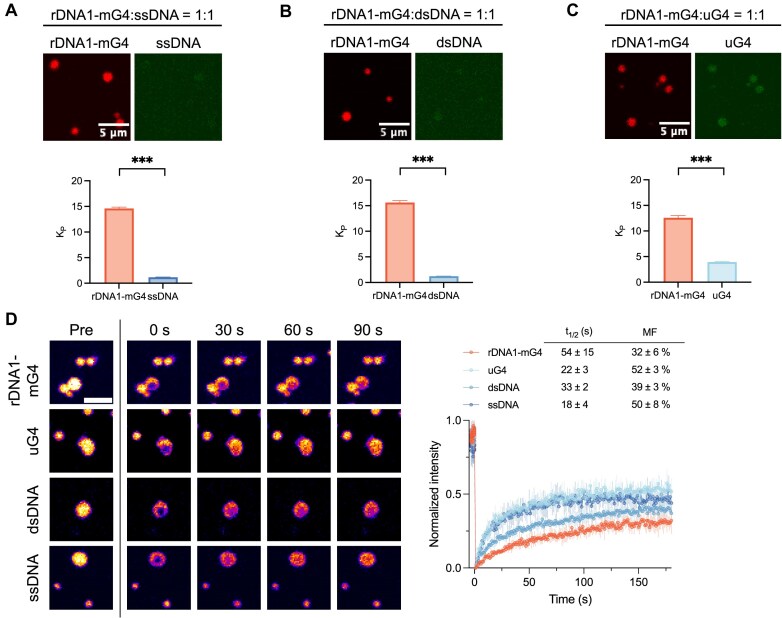
mG4 modulates the biophysical properties of the CSB droplets. rDNA1-mG4 is more readily sequestered into the CSB droplet when compared to ssDNA (**A**), dsDNA (**B**), and uG4 (**C**). Partitioning constant (*K*_P_) is defined as the ratio of fluorescence intensity inside/outside the droplets; statistical analysis was performed using Student’s *t*-test; ***P < .001; scale bar = 5 μm. Volume measurements for each condition were obtained from three field-of-view locations. Experiments were conducted with two independent preparations. (**D**) Left: Fluorescence confocal microscopy images, depicting the recovery of bleached Cy5-labeled oligonucleotides during FRAP measurement; scale bar = 5 μm. Right: FRAP recovery time and traces for different oligonucleotides, with their associated recovery half-time (*t*_1/2_) and mobile fraction (MF). Data obtained by 4–5 measurements for each condition.

The formation of biomolecular condensates leads to the uptake of specific substrates and the exclusion of others, which can be leveraged to regulate biological processes such as transcription [25, 48, 49]. Therefore, we next questioned whether the CSB droplet can preferentially uptake its binding partner, mG4, and segregate it from a mixture of other oligonucleotides. To assess this, we assembled CSB droplets at a 500 nM protein concentration in the presence of an equimolar mixture of fluorescently labeled mG4s and other non-mG4 oligonucleotides (250 nM) (Fig. 3A–C). Using confocal microscopy, we then calculated the partitioning coefficient (*K*_P_) for each oligonucleotide, defined as the ratio of fluorescence intensity within the droplet to that outside the droplet. Here, a higher fluorescence signal intensity within the droplet indicates that the oligonucleotide is selectively enriched in the condensate. As displayed in Fig. 3, CSB droplets can selectively recruit mG4 over single-stranded (ss)- and dsDNA, as well as uG4 (from the *c-MYC* sequence), yielding a partitioning coefficient *K*_P_ of ~15. ssDNA and dsDNA displayed a *K*_P_ ~1, indicating no partitioning into the CSB condensates (Fig. 3A and B). The uG4 (c-MYC) showed slightly higher partitioning into CSB droplets, with a *K*_P_ of around 3, which is accompanied by a modest decrease in the *K*_P_ of mG4 (Fig. 3C). Considering that CSB does not bind to uG4s [8], this partial partitioning might reflect the stacking of the uG4 on the mG4 scaffold, which allows it to be partially sequestered into the CSB droplet [26, 50]. Importantly, rDNA1-mG4 uptake appears to expel other non-mG4-forming DNA structures from the droplet, even though those structures can incorporate well into CSB droplets when alone (Fig. 2B).

To further validate the selective incorporation of rDNA1-mG4 into CSB-based droplets and exclusion of other DNA sequences, we normalized the lengths of the non-mG4 oligonucleotides to match that of rDNA1-mG4 (Supplementary Fig. S6A–C). We observed that the selective partitioning of rDNA1-mG4s is maintained even under this condition. Furthermore, we increased the molar excess of the non-mG4 oligonucleotides in the mixture to mimic a cellular context where dsDNA is in high excess. Pleasingly, we observed that selective incorporation of mG4 remains unaffected even at a five-fold higher concentration of ss- or dsDNA (Supplementary Fig. S7A–D), suggesting that the partitioning of non-mG4-forming oligonucleotides may be modulated upon mG4 binding.

To assess whether selective rDNA1-mG4 uptake into CSB droplets is a general feature across various mG4-forming sequences, we performed analogous partitioning experiments using rDNA2 and rDNA3 sequences. We also utilized mG4s from human CEB1 minisatellite [51] and hybrid DNA–RNA quadruplex (HQ) [52] sequences in the presence of ss- or dsDNA (CSB = 500 nM, oligonucleotides = 250 nM each). CEB1-mG4 and HQ-mG4 exhibit greater morphological differences than those between rDNA sequences, allowing us to further generalize the selective partitioning of mG4s into CSB. As expected, the partitioning behaviors of rDNA2-mG4 and rDNA3-mG4 are comparable to rDNA1-mG4; both display a *K*_P_ of around 23–25 and exclude ssDNA, dsDNA, and uG4 (Supplementary Fig. S8A–F). Both CEB1-mG4 and HQ-mG4 are also preferentially sequestered into CSB droplets, exhibiting *K*_P_ of ~9 and ~15 against ss-and dsDNA, respectively (Supplementary Fig. S9A–D). Altogether, these results suggest that mG4s promote phase separation of CSB, leading to their selective incorporation into the resulting droplets, while simultaneously expelling other non-mG4-forming DNA structures.

We next asked whether the incorporation of mG4s into CSB’s droplets alters their physicochemical properties, which might further underscore their relevance to the regulation of biological processes. We performed FRAP to assess the mobility of Cy5-labeled oligonucleotides within the droplet. FRAP revealed that mG4s had the lowest mobility among the tested oligonucleotides, with a half-time of recovery (*t*_1/2_) of rDNA1-mG4 = 53 ± 15 s (Fig. 3D), while rDNA2-mG4 and rDNA3-mG4 did not recover within the experimental timescale (Supplementary Fig. S10). Additionally, mG4-containing droplets also displayed the least amount of mobile fraction (rDNA1-mG4 = 32 ± 6%) compared to non-mG4 oligonucleotides (uG4 = 52 ± 3%, dsDNA = 39 ± 3%, ssDNA = 50 ± 8%). Indeed, CSB droplets containing the scrambled rDNA1s, which does not form mG4, exhibit a higher amount of mobile fraction (73 ± 8%) compared to its mG4-forming counterpart (Supplementary Fig. S10). These results indicate that mG4-containing droplets exhibit a more gel-like behavior, leading to decreased oligonucleotide mobility within the condensate. This further shows increased structural order conferred by mG4s within the condensate, which could originate from interchain crosslinking amongst strands that make up the mG4 [26, 53]. Additionally, this may indicate a greater potential of mG4-CSB condensates to sequester biomolecules under physiological conditions.

### Disruption of CSB–mG4 interaction abrogates droplet formation

**Figure 4.**
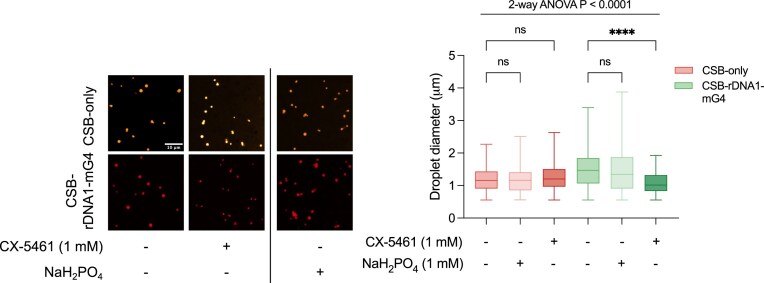
The addition of CX-5461 dissolves CSB-rDNA1-mG4 droplets, but not CSB-only droplets. Left: Confocal fluorescence microscopy images of CSB-only and CSB-rDNA1-mG4 droplets with and without 1 mM CX-5461; scale bar = 10 μm. Right: Quantification of the droplet diameter; statistical analysis was performed using two-way ANOVA with the Tukey correction; ^****^P < .0001, ns > 0.05.

Previous studies have shown that G4-stabilizing ligands can displace G4-binding proteins from their interaction with G4 motifs [8, 15]. Therefore, we asked whether introducing a G4 ligand could displace CSB bound to mG4s, causing dissolution of the corresponding condensates. We exposed a CSB-rDNA1-mG4 droplet (500 nM of each, pre-incubated at 30°C for 30 min) to 1 mM of the G4-ligand CX-5461 [54, 55], which we previously showed can displace CSB pre-bound to mG4s [8]. After 2-h incubation with CX-5461, we observed a substantial decrease in the droplets’ average apparent diameter (from 1.5 ± 0.5 μm to 1.1 ± 0.3 μm) (Fig. 4). In contrast, incubation of CSB-only droplets with CX-5461 caused no measurable changes in the droplets’ size or number (Fig. 4), indicating that the effect of the ligand is dependent on the presence of the mG4s in the droplets. Additionally, the vehicle used to solubilize the ligand (50 mM NaH_2_PO_4_, pH 4.0) does not affect the droplets in either case (Fig. 4). To further deconvolute whether the observed effect was caused by the structural features of the mG4 motif or by non-specific interaction with generic polyanions, we treated droplets formed in the presence of either ss- or dsDNA with the CX-5461 ligand (Supplementary Fig. S11A). Gratifyingly, no significant change in droplet size was observed upon ligand treatment (Supplementary Fig. S11B), further suggesting that protein displacement from the mG4s causes a reduction in droplet size.

Finally, to corroborate that preventing CSB from binding mG4 impairs droplet formation, we sought to block CSB’s mG4-binding domain by targeting its ATP-binding region, which overlaps with the mG4-binding domain [8]. To this end, we co-incubated CSB-rDNA1-mG4 (500 nM each) with 1 mM AppNHp, a non-hydrolyzable ATP analog. AppNHp addition led to a reduction in droplet size from 1.5 ± 0.5 μm to 1.0 ± 0.3 μm (Supplementary Fig. S12), which is comparable to that observed upon CX5461 treatment. This evidence indicates that AppNHp and rDNA1-mG4 compete for the same binding site and that CSB must remain available to bind mG4s for effective droplet formation. These results strongly suggest that CSB binding to mG4s leads to droplets with distinct physicochemical properties, which can be altered through small-molecule targeting. Altogether, our data indicate that CSB’s recognition of mG4s may be relevant to biological processes through the formation of biomolecular condensates.

## Discussion

In this work, we demonstrated that CSB can phase separate, and its binding selectivity towards mG4s can modulate this behavior and the physical properties of the biomolecular condensates formed. Importantly, we revealed that mG4s lower the saturation concentration required for droplet formation, a property not observed with any other oligonucleotides, suggesting a role for mG4s as a nucleation point for CSB phase separation. This hypothesis is further corroborated by our observations that CSB droplets exhibit greater resistance to environmental perturbations, such as elevated ionic strength, when bound to mG4s.

Notably, the selective binding of CSB to mG4s is also reflected in the selective uptake of mG4 and their reduced mobility within the droplet, which could be used to segregate genomic regions containing mG4s through CSB-based biomolecular condensates. This would agree with recent genomic studies indicating that G4 clusters involved with long-range DNA interactions (super-G4s) can be used to actively modulate transcription [15], facilitating chromatin contacts [56], and bridging enhancer–promoter interactions [15, 57–60]. Given the established relevance of both biomolecular condensates and G-quadruplex in transcriptional regulation, our findings strongly indicate that mG4s can be strategically formed within the genome to promote transcription through recruitment of CSB and modulation of biomolecular condensates formation. We anticipate that genome-wide mapping of mG4s and visualization of CSB condensates in live cells will be required to directly link the relevance of our findings in transcriptional regulation.

## Supplementary Material

gkag708_Supplemental_File

## Data Availability

The raw data underlying this article, including uncropped gel images, measurement of droplet area and their respective calculation of droplet diameter and/or volume, and raw FRAP traces, are available in Zenodo at https://doi.org/10.5281/zenodo.20936879.

## References

[1] Tiwari V, Baptiste BA, Okur MN et al. Current and emerging roles of Cockayne syndrome group B (CSB) protein. Nucleic Acids Res. 2021;49:2418–34. 33590097 10.1093/nar/gkab085 PMC7968995

[2] Stevnsner T, Muftuoglu M, Aamann MD et al. The role of Cockayne Syndrome group B (CSB) protein in base excision repair and aging. Mech Ageing Dev. 2008;129:441–8. 10.1016/j.mad.2008.04.009 18541289 PMC2538557

[3] Xu J, Lahiri I, Wang W et al. Structural basis for the initiation of eukaryotic transcription-coupled DNA repair. Nature. 2017;551:653–7. 10.1038/nature24658 29168508 PMC5907806

[4] Zhou D, Yu Q, Janssens RC et al. Live-cell imaging of endogenous CSB-mScarletI as a sensitive marker for DNA-damage-induced transcription stress. Cell Rep Methods. 2024;4:100674. 38176411 10.1016/j.crmeth.2023.100674 PMC10831951

[5] Xu J, Wang W, Xu L et al. Cockayne syndrome B protein acts as an ATP-dependent processivity factor that helps RNA polymerase II overcome nucleosome barriers. Proc Natl Acad Sci. 2020;117:25486–93. 32989164 10.1073/pnas.2013379117 PMC7568279

[6] van der Weegen Y, Golan-Berman H, Mevissen TET et al. The cooperative action of CSB, CSA, and UVSSA target TFIIH to DNA damage-stalled RNA polymerase II. Nat Commun. 2020;11:2014. 32355176 10.1038/s41467-020-15903-8 PMC7192910

[7] Laugel V, Dalloz C, Durand M et al. Mutation update for the CSB/ERCC6 and CSA/ERCC8 genes involved in Cockayne syndrome. Hum Mutat. 2010;31:113–26. 10.1002/humu.21154 19894250

[8] Liano D, Chowdhury S, Di Antonio M. Cockayne syndrome B protein selectively resolves and interact with intermolecular DNA G-quadruplex structures. J Am Chem Soc. 2021;143:20988–1002. 34855372 10.1021/jacs.1c10745

[9] Scheibye-Knudsen M, Tseng A, Jensen MB et al. Cockayne syndrome group A and B proteins converge on transcription-linked resolution of non-B DNA. Proc Natl Acad Sci USA. 2016;113:12502–7. 10.1073/pnas.1610198113 27791127 PMC5098674

[10] Parkinson GN, Lee MPH, Neidle S. Crystal structure of parallel quadruplexes from human telomeric DNA. Nature. 2002;417:876–80. 10.1038/nature755 12050675

[11] Valton AL, Prioleau MN. G-quadruplexes in DNA replication: a problem or a necessity?. Trends Genet. 2016;32:697–706. 10.1016/j.tig.2016.09.004 27663528

[12] Hänsel-Hertsch R, Beraldi D, Lensing SV et al. G-quadruplex structures mark human regulatory chromatin. Nat Genet. 2016;48:1267–72. 27618450 10.1038/ng.3662

[13] Spiegel J, Cuesta SM, Adhikari S et al. G-quadruplexes are transcription factor binding hubs in human chromatin. Genome Biol. 2021;22:117. 33892767 10.1186/s13059-021-02324-z PMC8063395

[14] Robinson J, Raguseo F, Nuccio SP et al. DNA G-quadruplex structures: more than simple roadblocks to transcription?. Nucleic Acids Res. 2021;49:8419–31. 34255847 10.1093/nar/gkab609 PMC8421137

[15] Robinson J, Flint G, Garner I et al. G-quadruplex structures regulate long-range transcriptional reprogramming to promote drug resistance in ovarian cancer cells. Genome Biol. 2025;26. 10.1186/s13059-025-03654-y PMC12255116 40646632

[16] Esain-Garcia I, Kirchner A, Melidis L et al. G-quadruplex DNA structure is a positive regulator of MYC transcription. Proc Natl Acad Sci USA. 2024;121:e2320240121. 38315865 10.1073/pnas.2320240121 PMC10873556

[17] Hänsel-Hertsch R, Simeone A, Shea A et al. Landscape of G-quadruplex DNA structural regions in breast cancer. Nat Genet. 2020;52:878–83. 10.1038/s41588-020-0672-8 32747825

[18] Komůrková D, Kovaříková AS, Bártová E. G-quadruplex structures colocalize with transcription factories and nuclear speckles surrounded by acetylated and dimethylated histones H3. Int J Mol Sci. 2021;22:1995. 33671470 10.3390/ijms22041995 PMC7922289

[19] David AP, Margarit E, Domizi P et al. G-quadruplexes as novel *cis*-elements controlling transcription during embryonic development. Nucleic Acids Res. 2016;44:4163–73. 10.1093/nar/gkw011 26773060 PMC4872077

[20] Le May N, Mota-Fernandes D, Vélez-Cruz R et al. NER factors are recruited to active promoters and facilitate chromatin modification for transcription in the absence of exogenous genotoxic attack. Mol Cell. 2010;38:54–66. 20385089 10.1016/j.molcel.2010.03.004

[21] Rippe K, Papantonis A. RNA polymerase II transcription compartments—from factories to condensates. Nat Rev Genet. 2025;26:775–88. 10.1038/s41576-025-00859-6 40537661

[22] Ryu K, Park G, Cho WK. Emerging insights into transcriptional condensates. Exp Mol Med. 2024;56:820–6. 10.1038/s12276-024-01228-9 38658705 PMC11059374

[23] Boija A, Klein IA, Sabari BR et al. Transcription factors activate genes through the phase-separation capacity of their activation domains. Cell. 2018;175:1842–55. 10.1016/j.cell.2018.10.042 30449618 PMC6295254

[24] Cho W-K, Spille J-H, Hecht M et al. Mediator and RNA polymerase II clusters associate in transcription-dependent condensates. Science (1979). 2018;361:412–5. 10.1126/science.aar4199 PMC6543815 29930094

[25] Alberti S . The wisdom of crowds: regulating cell function through condensed states of living matter. J Cell Sci. 2017;130:2789–96. 10.1242/jcs.200295 28808090

[26] Mimura M, Tomita S, Shinkai Y et al. Quadruplex folding promotes the condensation of linker histones and DNAs via liquid–liquid phase separation. J Am Chem Soc. 2021;143:9849–57. 10.1021/jacs.1c03447 34152774

[27] Vidal Ceballos A, Geissmann A, Favaro DC et al. RNA guanine content and G-quadruplex structure tune the phase behavior and material properties of biomolecular condensates. Sci Rep. 2025;15:9295. 40102453 10.1038/s41598-025-88499-y PMC11920403

[28] Yoo W, Song YW, Bansal V et al. G-quadruplex-dependent transcriptional regulation by molecular condensation in the Bcl3 promoter. Nucleic Acids Res. 2025;53:gkaf827. 40884402 10.1093/nar/gkaf827 PMC12397910

[29] Liu X, Xiong Y, Zhang C et al. G-quadruplex-induced liquid–liquid phase separation in biomimetic protocells. J Am Chem Soc. 2021;143:11036–43. 10.1021/jacs.1c03627 34270902

[30] Wang W, Li D, Xu Q et al. G-quadruplexes promote the motility in MAZ phase-separated condensates to activate CCND1 expression and contribute to hepatocarcinogenesis. Nat Commun. 2024;15:1045. 38316778 10.1038/s41467-024-45353-5 PMC10844655

[31] Wang Y, Cao K, Zong M et al. Mutual promotion of co-condensation of KRAS G-quadruplex and a well-folded protein HMGB1. Nucleic Acids Res. 2024;52:288–99. 10.1093/nar/gkad938 37897365 PMC10783520

[32] Hatos A, Tosatto SCE, Vendruscolo M et al. FuzDrop on AlphaFold: visualizing the sequence-dependent propensity of liquid–liquid phase separation and aggregation of proteins. Nucleic Acids Res. 2022;50:W337–44. 10.1093/nar/gkac386 35610022 PMC9252777

[33] Hardenberg M, Horvath A, Ambrus V et al. Widespread occurrence of the droplet state of proteins in the human proteome. Proc Natl Acad Sci USA. 2020;117:33254–62. 10.1073/pnas.2007670117 33318217 PMC7777240

[34] Vendruscolo M, Fuxreiter M. Sequence determinants of the aggregation of proteins within condensates generated by liquid–liquid phase separation: sequence code of aggregation in protein condensates. J Mol Biol. 2022;434:167201. 34391803 10.1016/j.jmb.2021.167201

[35] Campen A, Williams RM, Brown CJ et al. TOP-IDP-scale: a new amino acid scale measuring propensity for intrinsic disorder NIH public Access. Protein Peptide Letters. 2008;15:956–63. 18991772 10.2174/092986608785849164 PMC2676888

[36] Moses D, Yu F, Ginell GM et al. Revealing the hidden sensitivity of intrinsically disordered proteins to their chemical environment. J Phys Chem Lett. 2020;11:10131–6. 10.1021/acs.jpclett.0c02822 33191750 PMC8092420

[37] Theillet FX, Binolfi A, Frembgen-Kesner T et al. Physicochemical properties of cells and their effects on intrinsically disordered proteins (IDPs). Chem Rev. 2014;114:6661–714. 10.1021/cr400695p 24901537 PMC4095937

[38] Moses D, Ginell GM, Holehouse AS et al. Intrinsically disordered regions are poised to act as sensors of cellular chemistry. Trends Biochem Sci. 2023;48:1019–34. 10.1016/j.tibs.2023.08.001 37657994 PMC10840941

[39] Zheng T, Wake N, Weng SL et al. Molecular insights into the effect of 1, 6-hexanediol on FUS phase separation. EMBO J. 2025;44:2725–40. 10.1038/s44318-025-00431-2 40281357 PMC12084347

[40] Muzzopappa F, Hummert J, Anfossi M et al. Detecting and quantifying liquid–liquid phase separation in living cells by model-free calibrated half-bleaching. Nat Commun. 2022;13:7787. 36526633 10.1038/s41467-022-35430-y PMC9758202

[41] Krainer G, Welsh TJ, Joseph JA et al. Reentrant liquid condensate phase of proteins is stabilized by hydrophobic and non-ionic interactions. Nat Commun. 2021;12:1085. 33597515 10.1038/s41467-021-21181-9 PMC7889641

[42] Yuan X, Feng W, Imhof A et al. Activation of RNA polymerase I transcription by Cockayne syndrome group B protein and histone methyltransferase G9a. Mol Cell. 2007;27:585–95. 10.1016/j.molcel.2007.06.021 17707230

[43] Maharana S, Wang J, Papadopoulos DK et al. RNA buffers the phase separation behavior of prion-like RNA binding proteins. Science (1979). 2018;360:918–21. 10.1126/science.aar7366 PMC6091854 29650702

[44] Zhang H, Elbaum-Garfinkle S, Langdon EM et al. RNA controls PolyQ protein phase transitions. Mol Cell. 2015;60:220–30. 10.1016/j.molcel.2015.09.017 26474065 PMC5221516

[45] Sanchez-Burgos I, Espinosa JR, Joseph JA et al. RNA length has a non-trivial effect in the stability of biomolecular condensates formed by RNA-binding proteins. PLoS Comput Biol. 2022;18:e1009810. 35108264 10.1371/journal.pcbi.1009810 PMC8896709

[46] de Vries T, Novakovic M, Ni Y et al. Specific protein–RNA interactions are mostly preserved in biomolecular condensates. Sci Adv. 2024;10:eadm7435. 38446881 10.1126/sciadv.adm7435 PMC10917357

[47] Lebold KM, Best RB. Tuning formation of protein–DNA coacervates by sequence and environment. J Phys Chem B. 2022;126:2407–19. 10.1021/acs.jpcb.2c00424 35317553 PMC12442761

[48] Banani SF, Lee HO, Hyman AA et al. Biomolecular condensates: organizers of cellular biochemistry. Nat Rev Mol Cell Biol. 2017;18:285–98. 10.1038/nrm.2017.7 28225081 PMC7434221

[49] Banani SF, Rice AM, Peeples WB et al. Compositional control of phase-separated cellular bodies. Cell. 2016;166:651–63. 10.1016/j.cell.2016.06.010 27374333 PMC4967043

[50] Lech CJ, Heddi B, Phan AT. Guanine base stacking in G-quadruplex nucleic acids. Nucleic Acids Res. 2013;41:2034–46. 10.1093/nar/gks1110 23268444 PMC3561957

[51] Adrian M, Ang DJ, Lech CJ et al. Structure and conformational dynamics of a stacked dimeric G-quadruplex formed by the human CEB1 minisatellite. J Am Chem Soc. 2014;136:6297–305. 10.1021/ja4125274 24742225

[52] Zhang JY, Zheng KW, Xiao S et al. Mechanism and manipulation of DNA:RNA hybrid G-quadruplex formation in transcription of G-rich DNA. J Am Chem Soc. 2014;136:1381–90. 10.1021/ja4085572 24392825

[53] Majumder S, Coupe S, Fakhri N et al. Sequence-encoded intermolecular base pairing modulates fluidity in DNA and RNA condensates. Nat Commun. 2025;16:4258. 40335475 10.1038/s41467-025-59456-0 PMC12058984

[54] Xu H, Antonio D, M. MK et al. CX-5461 is a DNA G-quadruplex stabilizer with selective lethality in BRCA1/2 deficient tumours. Nat Commun. 2017;8:14432. 28211448 10.1038/ncomms14432 PMC5321743

[55] Sullivan HJ, Chen B, Wu C. Molecular dynamics study on the binding of an anticancer DNA G-quadruplex stabilizer, CX-5461, to human telomeric, C-Kit1, and c-myc G-quadruplexes and a DNA duplex. J Chem Inf Model. 2020;60:5203–24. 10.1021/acs.jcim.0c00632 32820923

[56] Flynn SM, Dhir S, Herka K et al. Improved simultaneous mapping of epigenetic features and 3D chromatin structure via ViCAR. Genome Biol. 2024;25:237. 39227991 10.1186/s13059-024-03377-6 PMC11370281

[57] Lyu J, Shao R, Kwong Yung PY et al. Genome-wide mapping of G-quadruplex structures with CUT&tag. Nucleic Acids Res. 2022;50:E13. 34792172 10.1093/nar/gkab1073 PMC8860588

[58] Williams JD, Houserova D, Johnson BR et al. Characterization of long G4-rich enhancer-associated genomic regions engaging in a novel loop:loop ‘G4 kissing’ interaction. Nucleic Acids Res. 2020;48:5907–25. 10.1093/nar/gkaa357 32383760 PMC7293029

[59] Roy SS, Bagri S, Vinayagamurthy S et al. Artificially inserted strong promoter containing multiple G-quadruplexes induces long-range chromatin modification. eLife. 2024;13:RP96216. 39158543 10.7554/eLife.96216 PMC11333042

[60] Antariksa NF, Di Antonio M. The emerging roles of multimolecular G-quadruplexes in transcriptional regulation and chromatin organization. Acc Chem Res. 2024;57:3397–406. 10.1021/acs.accounts.4c00574 39555660 PMC11618987

