# Supplementary material for: Recognition of multimolecular G-quadruplex regulates phase separation of cockayne syndrome B
Source: Nucleic Acids Res. 2026 Jul 15;54(13):gkag708. doi: 10.1093/nar/gkag708 (PMC13369332; doi:10.1093/nar/gkag708)
Supplement: gkag708_Supplemental_File [file gkag708_supplemental_file.docx]

Recognition of Multimolecular G-Quadruplex Regulates Phase Separation of Cockayne Syndrome B

Naura Fakhira Antariksa,^1,2^ Michele Stasi,^1,2^ Marco Di Antonio*^1,2,3^

^1^Department of Chemistry, Molecular Sciences Research Hub, Imperial College London, W12 0BZ, UK

^2^Institute of Chemical Biology, Molecular Sciences Research Hub, Imperial College London, W12 0BZ, UK

^3^The Francis Crick Institute, 1 Midland Road, London NW1 1AT, UK

**Electronic Supporting Information**

## Table of Contents

Table of Contents ……………………………………………………………………………………………………………… 2

Table S1. Sequences for oligonucleotides …………………………………………………………………………… 3

Figure S1. CSB phase separation is affected by salt titration, but not 1,6-hexanediol ……… 4

Figure S2. CSB-labeling with Cy3 ………………………………………………………………………………….…… 5

Figure S3. CSB droplet can recruit mG4 structures folded from ribosomal DNA sequences ……… 6

Figure S4. Titration of oligonucleotides at 100 nM CSB ……………………………………………………. 7

Figure S5. KCl titration of other oligonucleotides ………………………………….……………………………. 8

Figure S6. Upon normalization of ssDNA, dsDNA, and uG4 lengths, rDNA1-mG4 is still preferentially sequestered ………………………………………………………………………………………………….. 9

Figure S7. CSB droplets specifically sequester rDNA1-mG4 even at a higher molar ratio of non-mG4 species ………………………………………………………………………………………………………………………… 10

Figure S8. Other rDNA mG4 sequences are also preferentially sequestered into CSB droplets 11

Figure S9. CSB droplets selectively uptake non-rDNA mG4-forming sequences ..……………… 12

Table S2. Number of droplets identified in each partitioning condition …………………………… 13

Figure S10. FRAP traces of other oligonucleotides ……………………………………………………………… 14

Figure S11. CSB droplets with non-mG4 oligonucleotides are unaffected by the addition of the G4-stabilizing ligand, CX-5461………………………………………………………………………………………………… 15

Figure S12. The addition of AppNHp dissolves CSB-mG4 droplets ………………………………………. 16

| **Oligonucleotide** | **Used in** | **Sequence (5’-3’)** |
| --- | --- | --- |
| (5’-Cy5)-rDNA1 multimolecular G4 | Oligo titration, KCl titration, Partitioning, FRAP, Gel analysis | /5Cy5/ATAATTATAAATAAATAATAGGGGCCGGGGGTGGGGTCGGCGGGGAAA |
| (5’-Cy5)-rDNA2 multimolecular G4 | Oligo titration, KCl titration, Partitioning, FRAP, Gel analysis | /5Cy5/ATAATTATAAATAAATAATAGGGTCGGGGGGTGGGGCCCGGGCCGGGG |
| (5’-Cy5)-rDNA3 multimolecular G4 | Oligo titration, KCl titration, Partitioning, FRAP, Gel analysis | /5Cy5/ATAATTATAAATAAATAATAGGGAGGGAGACGGGGGGG |
| (5’-FAM)-*c-MYC* unimolecular G4 | Oligo titration, KCl titration, Partitioning, FRAP, Gel analysis | /5FAM/TGAGGGTGGGTAGGGTGGGTAA |
| dsDNA (strand 1) | Oligo titration, KCl titration, Partitioning, FRAP | TTTTTTAAGAGCGTTAAGAGCGTGAAC |
| (5’-Cy3)-dsDNA (strand 2) | Oligo titration, KCl titration, Partitioning, FRAP | /5Cy3/CCTTGTTCACGCTCTTAACGCTCTTAAAAAA |
| (5’-Cy3)-ssDNA | Oligo titration, KCl titration, Partitioning, FRAP | /5Cy3/CCTTGTTCACGCTCTTAACGCTCTTAAAAAA |
| (5’-FAM)-*c-MYC* unimolecular G4 | Partitioning (Fig. S7, S8) | /5FAM/ATAATTATAAATAAATAATATTTTTGGGGAGGGTGGGGAGGGTGGGGAAGG |
| dsDNA (strand 1) | Partitioning (Fig. S7, S8) | ATAATTATAAATAAATAATATTTTTTAAGAGCGTTAAGAGCGTGAACAAGG |
| (5’-FAM)-dsDNA (strand 2) | Partitioning (Fig. S7, S8) | /56-FAM/CCTTGTTCACGCTCTTAACGCTCTTAAAAAATATTATTTATTTATAATTAT |
| (5’-FAM)-ssDNA | Partitioning (Fig. S7, S8) | /56-FAM/ ATAATTATAAATAAATAATATTTTTTAAGAGCGTTAAGAGCGTGAACAAGG |
| (5’-Cy5)-Scrambled rDNA1 | KCl titration, Partitioning, Gel analysis, FRAP, Gel analysis | /5Cy5/TAGGATGAGAGGATGAGGAGACGTAGACATTGTCGTAGGGCAATAGAG |
| (5’Cy5)-CEB1 stacked dimeric G4 | Partitioning | /5Cy5/AGGGGGGAGGGAGGGTGG |
| Hybrid quadruplex RNA G-core | Partitioning | GAGUUGUUGAUUGAGUCGCUGACAGGGGGGGAGGGGGA |
| Hybrid quadruplex DNA G-core-(3’-Cy5) | Partitioning | GGGGGAGGGGGGGAGTCAGCGACTCAATCAACAACTC/3Cy5Sp/ |
| 93-del bimolecular mG4 | Gel analysis | GGGGTGGGAGGAGGGTATAATTATAAATAAATAATA/36-FAM/ |

**Table S1** Sequences for oligonucleotides used in this work

**Figure S1. CSB phase separation is affected by salt titration, but not 1,6-hexanediol**. Progressive drop of A) average droplet diameter and B) number of droplets under increasing KCl concentration shown at three CSB concentrations (250-1000 nM). Error bars are shown for the standard deviation. C) Titration of 1,6-hexanediol (0-5% v/v) does not affect CSB droplet formation. Scale bar = 5 μm. Average droplet calculation and number of droplets for each condition was obtained from three field-of-views.

**Figure S2. CSB-labeling with Cy3**. Cy3-labeled CSB was run on SDS-PAGE, where the in-gel fluorescence (left) was visualized using LI-COR Odyssey**®** M imaging system before Coomassie Brilliant Blue staining (right). Unlabeled CSB MW = 170.9 kDa. Protein concentration post-labeling was estimated from the BSA curve (MW = 66 kDa).

**Figure S3. CSB droplet can recruit mG4 structures folded from ribosomal DNA sequences**. A) Left: gel image of rDNA sequences (diluted to 500 nM after annealing at the indicated concentrations to mimic microscopy sample preparation) visualized by SYBRGold dye, confirming that these sequences fold into multimeric species under microscopy experimental conditions. Right: NMM staining of the same gel, indicating the mG4 species. B) Confocal fluorescence microscopy images showing droplet formation in a CSB-only mixture and CSB-rDNA-mG4 mixtures. Scale bar = 5 μm.

**Figure S4. Titration of oligonucleotides at 100 nM CSB**. A) Top: Confocal fluorescence microscopy images showing the effect of adding rDNA2-mG4 and rDNA3-mG4 to CSB (100 nM); scale bar = 10 μm. Bottom: Quantification of total droplet volumes. Red boxes denote the oligonucleotide concentration with the highest amount of droplet material (rDNA2-mG4 = 25 nM, rDNA3-mG4 = 10 nM). Scale bar = 10 μm. B) Confocal fluorescence microscopy images depicting the titration of Myc uG4 and dsDNA to 100 nM CSB. Red boxes denote the oligonucleotide concentration with the highest amount of droplet material (uG4 = 100 nM, dsDNA = 50 nM). Scale bar = 10 μm.

**Figure S5. KCl titration of other oligonucleotides.** Left: KCl titration of mG4 oligonucleotides (rDNA2-mG4 and rDNA3-mG4) and scrambled rDNA1 sequence (rDNA1s) at 500 nM equimolar concentrations of CSB and each respective oligonucleotide; scale bar = 10 μm. Right: Quantification of the differential drop in droplet volume (V_0_ = total droplet volume at 100 mM KCl, V_i_ = total droplet volume at a given KCl concentration) amongst CSB-oligonucleotide condensates.

**Figure S6. Upon normalization of ssDNA, dsDNA, and uG4 lengths, rDNA1-mG4 is still preferentially sequestered**. Confocal fluorescence microscopy images depicting the partitioning of oligonucleotides into the CSB droplet at a 1:1 ratio of A) rDNA1-mG4:ssDNA, B) rDNA1-mG4:dsDNA, and C) rDNA1-mG4uG4. Protein concentration is 500 nM; 1:1 oligonucleotide ratio = 250 nM : 250 nM. Scale bar = 5 μm. Statistical analysis was performed using Student’s t-test; *** P < 0.001.

**Figure S7. CSB droplets specifically sequester rDNA1-mG4 even at a higher molar ratio of non-mG4 species**. Confocal fluorescence microscopy images depicting the partitioning of oligonucleotides into the CSB droplet at a 1:3 ratio of A) rDNA1-mG4:ssDNA and B) rDNA1-mG4:dsDNA, and a 1:5 ratio of C) rDNA1-mG4:ssDNA, and D) rDNA1-mG4:dsDNA. Protein concentration is 100 nM; 1:3 oligonucleotide ratio = 12.5 nM : 37.5 nM; 1:5 oligonucleotide ratio = 12.5 nM : 62.5 nM. Scale bar = 5 μm. Statistical analysis was performed using Student’s t-test; ** P < 0.01.

**Figure S8. Other rDNA mG4 sequences are also preferentially sequestered into CSB droplets**. Confocal fluorescence microscopy images depicting the partitioning of oligonucleotides into the CSB droplet at a 1:1 ratio of rDNA2-mG4 with A) ssDNA, B) dsDNA, and C) uG4 and rDNA3-mG4 with D) ssDNA, E) dsDNA, and F) uG4. Protein concentration is 500 nM; oligonucleotide concentration is 250 nM each. Scale bar = 5 μm. Statistical analysis was performed using Student’s t-test; **** P < 0.0001, *** P < 0.001, ** P < 0.01.

**Figure S9. CSB droplets selectively uptake non-rDNA mG4-forming sequences**. Confocal fluorescence microscopy images depicting the partitioning of oligonucleotides into the CSB droplet at a 1:1 ratio of A) CEB1-mG4:ssDNA, B)HQ-mG4:ssDNA, C) CEB1-mG4:dsDNA, and D) HQ-mG4:dsDNA. Protein concentration is 500 nM; 1:1 oligonucleotide ratio = 250 nM : 250 nM. Scale bar = 5 μm. Statistical analysis was performed using Student’s t-test; **** P < 0.0001, *** P < 0.001, ** P < 0.01.

| **Oligonucleotide Mixture** | **Concentration Ratio** | **Number of droplets** |
| --- | --- | --- |
| rDNA1 vs ssDNA | 1:1 (250 nM : 250 nM) | 424 |
| rDNA1 vs dsDNA | 1:1 (250 nM : 250 nM) | 208 |
| rDNA1 vs uG4 | 1:1 (250 nM : 250 nM) | 249 |
| rDNA2 vs ssDNA | 1:1 (250 nM : 250 nM) | 1371 |
| rDNA2 vs dsDNA | 1:1 (250 nM : 250 nM) | 812 |
| rDNA2 vs uG4 | 1:1 (250 nM : 250 nM) | 729 |
| rDNA3 vs ssDNA | 1:1 (250 nM : 250 nM) | 1102 |
| rDNA3 vs dsDNA | 1:1 (250 nM : 250 nM) | 898 |
| rDNA3 vs uG4 | 1:1 (250 nM : 250 nM) | 489 |
| rDNA1 vs ssDNA | 1:3 (12.5 nM : 37.5 nM) | 277 |
| rDNA1 vs dsDNA | 1:3 (12.5 nM : 37.5 nM) | 212 |
| rDNA1 vs ssDNA | 1:5 (12.5 nM : 62.5 nM) | 294 |
| rDNA1 vs dsDNA | 1:5 (12.5 nM : 62.5 nM) | 248 |
| CEB1 vs ssDNA | 1:1 (250 nM : 250 nM) | 327 |
| CEB1 vs dsDNA | 1:1 (250 nM : 250 nM) | 339 |
| HQ vs ssDNA | 1:1 (250 nM : 250 nM) | 659 |
| HQ vs dsDNa | 1:1 (250 nM : 250 nM) | 368 |

**Table S2.** Number of droplets identified in each partitioning condition.

**Figure S10. FRAP traces of other oligonucleotides.** Left: Fluorescence confocal microscopy images, depicting the recovery of bleached Cy5-labeled oligonucleotides during FRAP measurement; scale bar = 5 μm. Right: FRAP recovery time and traces for different oligonucleotides, with their associated recovery half-time (t_1/2_) and mobile fraction (MF). Data obtained by 5 measurements for each condition.

**Figure S11. CSB droplets with non-mG4 oligonucleotides are unaffected by the addition of the G4-stabilizing ligand, CX-5461**. A) Confocal fluorescence microscopy images depicting the CSB-dsDNA and CSB-ssDNA droplets with and without 1 mM CX-5461; scale bar = 10 μm. B) Quantification of the droplet diameter; statistical analysis was performed using 2-way ANOVA with the Tukey correction; ns > 0.05.

**Figure S12. The addition of AppNHp dissolves CSB-mG4 droplets**. A) Confocal fluorescence microscopy images depicting the CSB-dsDNA and CSB-ssDNA droplets with and without 1 mM AppNHp. B) Quantification of the droplet diameter; statistical analysis was performed using Student’s t-test; ****P < 0.0001. C) Representative EMSA gel showing that increasing AppNHP addition displaces CSB from rDNA1-mG4.
